# Computing minimal nutrient sets from metabolic networks via linear constraint solving

**DOI:** 10.1186/1471-2105-14-114

**Published:** 2013-03-27

**Authors:** Steven Eker, Markus Krummenacker, Alexander G Shearer, Ashish Tiwari, Ingrid M Keseler, Carolyn Talcott, Peter D Karp

**Affiliations:** 1 Computer Science Laboratory, SRI International, Menlo Park, CA 94025, USA; 2 Bioinformatics Research Group, SRI International, Menlo Park, CA 94025, USA

**Keywords:** Binary decision diagrams, Computational biology, Linear constraint solving, Minimal nutrient sets, SMT solvers, Metabolic and regulatory networks, Cellular metabolism

## Abstract

**Background:**

As more complete genome sequences become available, bioinformatics challenges arise in how to exploit genome sequences to make phenotypic predictions. One type of phenotypic prediction is to determine sets of compounds that will support the growth of a bacterium from the metabolic network inferred from the genome sequence of that organism.

**Results:**

We present a method for computationally determining alternative growth media for an organism based on its metabolic network and transporter complement. Our method predicted 787 alternative anaerobic minimal nutrient sets for *Escherichia coli* K–12 MG1655 from the EcoCyc database. The program automatically partitioned the nutrients within these sets into 21 equivalence classes, most of which correspond to compounds serving as sources of carbon, nitrogen, phosphorous, and sulfur, or combinations of these essential elements. The nutrient sets were predicted with 72.5% accuracy as evaluated by comparison with 91 growth experiments. Novel aspects of our approach include (a) exhaustive consideration of all combinations of nutrients rather than assuming that all element sources can substitute for one another(an assumption that can be invalid in general) (b) leveraging the notion of a machinery-duplicating constraint, namely, that all intermediate metabolites used in active reactions must be produced in increasing concentrations to prevent successive dilution from cell division, (c) the use of Satisfiability Modulo Theory solvers rather than Linear Programming solvers, because our approach cannot be formulated as linear programming, (d) the use of Binary Decision Diagrams to produce an efficient implementation.

**Conclusions:**

Our method for generating minimal nutrient sets from the metabolic network and transporters of an organism combines linear constraint solving with binary decision diagrams to efficiently produce solution sets to provided growth problems.

## Background

Approximately 75% of microbial organisms are unculturable (cannot be grown in the laboratory) even as we can fully sequence their genomes [1,2]. Determination of proper laboratory growth conditions presents a significant barrier to a comprehensive understanding of the microbial world.

Given the high cost of evaluating laboratory growth conditions and the relative abundance of powerful genome sequencing resources, it makes sense to ask whether we can use the metabolic network inferred from an organism’s genome sequence to predict the media that will support the growth of the organism. We have previously shown that the biochemical reactions and metabolic pathways of an organism can be inferred from its annotated genome [3-5]. We have also shown that the completeness of a metabolic network can be evaluated using a “forward propagation” approach [6]. This purely qualitative modeling approach treats each reaction as a rule that will “fire” if all of its reactants are present. When a reaction fires, its products are added to the metabolite pool. This process is then repeated using the new, larger metabolite pool, until no more reactions fire. For example, a model of the *Escherichia coli* metabolic network could be “fed” the constituent compounds of M9 minimal medium, and the expectation would be that all the biomass compounds should be present in the final, fixed set of compounds generated via forward propagation.

This qualitative analysis method is a good starting point for deriving minimal nutrient sets, but it has a major limitation. It treats the organism as an empty factory lacking everything except the provided nutrients. But cells do not start as empty bags of metabolites — they contain a wide variety of compounds that “prime the pump” for their own syntheses — *“Omnis cellula e cellula”* (*“Every cell from another cell”* — Francois-Vincent Raspail) [7]. Consequently, the forward propagation approach cannot properly analyze cycles in which an organism begins with some amount of a compound *C* and uses *C* in combination with other nutrients to generate more *C*. Such cycles do occur in practice (e.g., glycolysis consumes ATP before producing ATP). Modeling these cycles requires the handling of stoichiometric reactions and the tracking of relative rates of production and consumption of compounds, and is addressed herein.

Flux-Balance Analysis (FBA) methods can also be used to predict whether a given nutrient set supports growth. However, to permit computational tractability, FBA approaches begin with a starting “seed” medium and generate new media in which only one nutrient at a time is changed, to vary the source of one element, e.g., nitrogen. Thus, the method does not evaluate all combinations of nutrients — it assumes that if a given nitrogen source supports (or does not support) growth with one carbon source, it will exhibit the same behavior for all carbon sources (or other element sources). We show that this assumption of orthogonality of element sources is not guaranteed to hold, and argue that metabolic network algorithms should be designed to analyze networks with unusual properties, or we take the risk of finding only those nutrient sets that our algorithms expect to see.

We address the challenge of predicting growth media from genome data by developing a novel constraint-based algorithm that infers minimal nutrient sets for an organism based on its metabolic network. The algorithm requires (1) a set R of metabolic reactions for the organism, (2) a set of *transportables*T that are potential nutrients (inferred from the transporter proteins of the organism), and (3) a set of *biomass compounds*B that must be produced for growth. A subset N⊆T of transportables is a *nutrient set* if the set B is *producible* from *N* where producible may have different definitions, depending on assumptions. A nutrient set *N* is minimal if no proper subset of *N* is a nutrient set. In other words, a nutrient set *N* is a minimal nutrient set if we cannot form a new nutrient set by removing one or more compounds from *N*.

Because our algorithm sometimes infers thousands of minimal nutrient sets, which are difficult to comprehend and to evaluate, we also developed an algorithm that computes nutrient *equivalence classes* from minimal nutrient sets. Two nutrients *A* and *B* belong to an equivalence class if for every minimal nutrient set containing *A* there also exists a minimal nutrient set in which *B* is substituted for *A*, and vice versa. In *E. coli* we find that these equivalence classes often correspond to compounds that supply a given element (e.g., carbon sources). We can communicate all computed minimal nutrient sets to the user more effectively by presenting the nutrient equivalence classes, plus a reduced set of minimal nutrient sets in which we retain only those minimal nutrient sets that contain one representative from each equivalence class.

We apply our algorithm to *E. coli* by using the manually curated reaction network stored in EcoCyc [8] and validate the algorithm by comparison with prior Phenotype Microarray data.

## Methods

### The prediction pipeline

**Figure 1:**
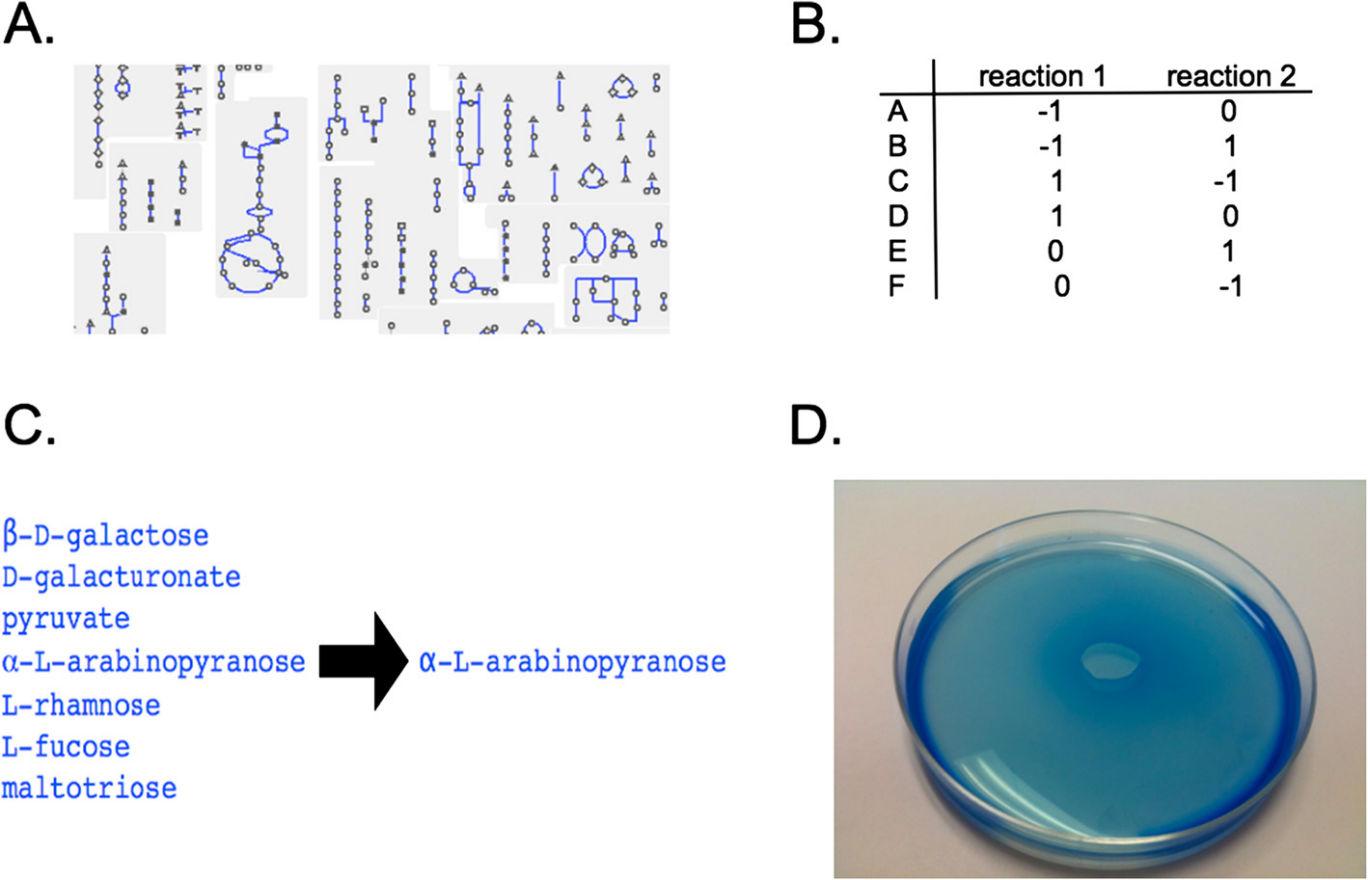
**Testable nutrient predictions are generated from metabolic network data.** Our prediction method operates via a four-step process. (**A**) A metabolic reaction network can be obtained from manual curation, computational inference, or a combination thereof. (**B**) The reaction network is converted into a constraint problem and solved for minimal nutrient sets. (**C**) These minimal nutrient sets are distilled into easier-to-handle “equivalence classes”: compounds *A* and *B* are in the same equivalence classes if for every nutrient set including *A*, an equivalent nutrient set exists with *B* substituted for *A*. (**D**) The equivalence classes are then evaluated by comparison with laboratory experiments.

The pipeline from metabolic network to evaluated results proceeds via four steps (Figure 1). First, we define a constraint-based model (Step 1). This model is then solved to identify minimal nutrient sets (Step 2). These minimal nutrient sets are then distilled into a smaller and easier-to-evaluate set of compound equivalence classes (Step3). Finally, these equivalence classes are evaluated by comparing them to previous experimental growth data and laboratory growth experiments (Step 4).

### Building constraint-based models

Our starting point is the organism’s metabolic network. We analyze the properties of this network by using a constraint-based approach. These constraints are expressed over the flux of the reactions in the network. We describe the method for generating constraints from the metabolic network below in two parts. First, we build a naïve steady-state model that allows metabolites that are in neither the nutrient set nor the biomass set to have zero net production. Second, we show why this naïve, steady-state model is an unrealistic model of growing and dividing cells and then propose a more sophisticated model that can be shown to be more accurate by using a purely molecule-counting argument. This more sophisticated model (which we call the Machinery-Duplicating Model) is what we then use for our predictions.

#### The steady-state model

We start with the following hypothetical metabolic network:

##### 

**Example 1. ***Let*R*consist of the two unidirectional reactions:*

(1) $$\begin{array}{ll} A+B\to C+D & \; \end{array}$$

(2) $$\begin{array}{ll} C+F\to B+E & \; \end{array}$$

*Let*B={E}*(i.e.Eis the sole biomass compound).*

Suppose *A* and *F* are available as nutrients. Using forward propagation, neither of the reactions can fire because both *B* and *C* are unavailable. However, we can assume more realistically that the cell is not an empty bag and that *n* molecules of *B* are initially available. Then reaction (1) could fire *n* number of times, creating *C*, which could be used to fire reaction (2) *n* times recreating the *n* molecules for *B*. Within this framework, we are no longer reasoning about a monotonically increasing set of compounds, but instead about relative reaction rates and the rate of the net production or consumption of compounds.

The reactions above can be written as a *stoichiometric matrixM* in Table 1.

**Table 1:** A stoichiometric matrix in which each row represents one metabolite and each column represents one reaction

	**Reaction 1**	**Reaction 2**
A	−1	0
B	−1	1
C	1	−1
D	1	0
E	0	1
F	0	−1

Here, *M*_*i*,*j*_ records the net production (negative for consumption) of the *i*th compound by the *j*th reaction. We represent the rates of the reactions or *flux* by the column vector of variables *r*=[*r*_1_,*r*_2_]^*T*^ (using the transpose convention for representing column vectors), where *r*_1_ is the rate of reaction (1) and *r*_2_ is the rate of reaction (2). The rate of production of compounds by the system is given by the column vector *p*=*Mr*.

Given a putative nutrient set *N* and a set B of biomass compounds, we place constraints on the compound production rates (entries of *p*), as follows: 

1. If the *i*th compound is in B and not in *N* then we require *p*_*i*_>0.

2. If the *i*th compound is not in B and not in *N* then we require *p*_*i*_≥0.

In our example B={E} and *N*={*A*,*F*}. The compound *B* is consumed by reaction (1) with rate *r*_1_ and created by reaction (2) with rate *r*_2_ so it has a net production of −*r*_1_+*r*_2_ and thus *B* yields a constraint:

$$-r_{1}+r_{2}\geq 0.$$

Similar analysis yields the constraints

$$\begin{array}{rr} r_{1}-r_{2}\geq 0 & \;\\ r_{1}\geq 0 & \;\\ r_{2}>0 & \; \end{array}$$

for compounds *C*, *D*, and *E*, respectively.

Because reactions are not allowed to run in reverse, we must add the additional constraints that *r*_1_≥0 and *r*_2_≥0. We say that *N* is a *steady-state* nutrient set if there exists a vector *r* that satisfies the above constraints. In our example, *r*_1_=*r*_2_=*k* for any *k*>0 satisfies all the constraints. All the generated constraints are linear; thus, checking whether N⊆T is a steady-state nutrient set reduces to checking the feasibility of a linear program.

Based on a simple molecule-counting argument and linear algebra, we make the following claim relating the steady-state model to experimental observations.^a^

##### 

**Claim 1. ***Assume the set*R*includes all reactions available to the organism. This set may also include extraneous reactions that are not actually available to the organism, due to errors in the available data. Assume that set*B*only contains compounds that the organism must produce to grow (this set need not, however, be complete). Then the steady-state model over-approximates observable behaviors in the following sense: If the steady-state model predicates that some set*N⊂T*of transportables is not a nutrient set then organism will be unable to grow on nutrient setNin the laboratory.*

##### 

**Justification 1.** For a contradiction, suppose we observe our organism to grow on *N* in the laboratory. Because everything in B must be produced by the organism and it has only the reactions in R and the nutrients in *N* at its disposal, it must have found a set of fluxes for R that yield positive net production of each compound in *B* and non-negative net production of each compound not in *N*. However, because our system of linear constraints does not have a solution with putative nutrient set *N*, such set of fluxes does not exist.

Notice that although we need the set T of transportables in order to form putative nutrient sets, the critical parameters of our model are the set R of reactions and the set B of biomass compounds. For a pair 〈R,B〉, we call the assumption that R includes at least all reactions available to the organism and B contains only compounds that the organism must produce to grow the *perfect data assumption*. Though possibly unrealistic in practice, unless we are studying modeling methods that explicitly model errors and omissions in the data, making formal comparisons without an assumption of this kind is difficult on paper.

Informally, Claim 1 says that under the perfect data assumption, the steady-state model can produce only one-sided errors: false positives. If it predicts growth on a putative nutrient set *N* then although there exists a flux that produces B, growth may not be observed in the laboratory for a number of reasons including negative interactions such as toxicity, competitive reactions, or gene regulation that we do not attempt to model. But under the perfect data assumption, false negatives are impossible; if the model predicts failure to grow on a putative nutrient set *N* then it is *arithmetically impossible* for the organism to grow on *N*. However if growth is indeed observed in the laboratory, then barring experimental error, at least one of our initial assumptions about the completeness of R or the necessity of producing all the compounds in B must be incorrect.

#### The machinery-duplicating model

The steady-state model described above is somewhat unsatisfactory. We have assumed a set B of compounds as a proxy for growth. However, if a growing cell eventually divides into two daughter cells that are identical^b^ to the original cell, then all of the intermediate metabolites that were used along active pathways to produce compounds of B from putative nutrients *N* must also be duplicated (we do not say anything additional about intermediate metabolites that arise only on inactive pathways); in essence a dividing cell must at the very least be able to duplicate the active part of its metabolic machinery in addition to producing B.

Informally, we account for the need to duplicate the active part of the metabolic machinery by adding additional constraints to the steady-state model to require that if a compound *C* is used as a reactant in a reaction with nonzero flux and C∉N∪B then *C* must have strictly positive net production, thus explicitly requiring that more of *C* will be produced as the organism grows and divides.

How we frame this constraint mathematically is rather subtle. Suppose for some compound $C_{j}\notin N\cup B$, the set of indices of reactions that use it as a reactant is *I*_*j*_. Then for a given flux *r*, *C* is clearly used if and only if there exists *i*∈*I*_*j*_ such that *r*_*i*_>0. Since we have constrained the rates to be non-negative, that is equivalent to the test $\underset{i\in I_{j}}{\sum }r_{i}>0$. This suggests formulating a constraint in terms of the sum of reaction rates,

$$s_{j}=\underset{i\in I_{j}}{\sum }r_{i}.$$

Suppose the net production of *C*_*j*_ is given by the linear combination *p*_*j*_. We would like to require *p*_*j*_ to be strictly positive whenever *s*_*j*_ is strictly positive. The question is how to frame this as a linear constraint.

One approach is to require that *p*_*j*_≥*αs*_*j*_ for some fudge factor *α*, thus constraining *p*_*j*_ to be strictly positive when *s*_*j*_ is strictly positive. The problem is in determining what fudge factor *α* to use, since too large a value might lead to an unsatisfiable constraint even though there exists a flux producing a positive amount of *C*_*j*_.

Requiring *p*_*j*_≥*s*_*j*_/(*s*_*j*_+1) would work because *s*_*j*_/(*s*_*j*_+1) is always less than one and thus any flux producing a positive amount of *C*_*j*_ can be “scaled up” to satisfy this constraint. Unfortunately, however, when multiplied out, this constraint turns out to be quadratic.

Our solution is to relax the requirement that our linear system be a conjunction of linear constraints and instead allow a monotone Boolean combination of linear constraints. Thus, we add the constraint

(3) $$\left(p_{j}>0\right)\vee \underset{i\in I_{j}}{\wedge }\left(r_{i}=0\right)$$

That is, either production of *C*_*j*_ is positive, or all the reactions that use it as a reactant have zero rate. We call such constraints “make it if you use it” constraints. A nutrient set solution that satisfies these additional constraints will be called a *machinery-duplicating nutrient set*.

Notice that the system of linear constraints produced by this new model is not a simple conjunction of linear inequalities but a more general Boolean combination of linear inequalities. Checking the *feasibility* (or synonymously, the *satisfiability*^c^) of such systems falls beyond the capabilities of a regular Linear Programming (LP) package and instead requires the use of a more recent development in computer science called a *Satisfiability Modulo Theories (SMT) solver*[9]. This newer kind of solver has the added advantage of working with exact (rational number) arithmetic that sidesteps the issues of round-off error and numerical stability. These issues can be a problem with Linear Programming packages that typically use inexact floating-point arithmetic.

As with the steady-state model, we claim that our more sophisticated machinery-duplicating model over-approximates observable behavior and justify the claim with a molecule-counting argument.

##### 

**Claim 2. ***Given a pair*〈R,B〉*that satisfy the perfect data assumption for some organism, if the machinery-duplicating model predicates that some set*N⊂T*of transportables is not a nutrient set then the organism will be unable to grow on nutrient setNin the laboratory.*

##### 

**Justification 2.** For a contradiction, suppose we observe our organism to be growing on *N* in the laboratory. Thus, the cells must be dividing, and the metabolic machinery that is active to produce B from *N* must itself be duplicated. The growing colony of cells must, considered as a single system, have found a set of fluxes for R that yield positive amounts of each compound in B*and* for the reactants of every reaction with nonzero flux that are not members of *N*. However, because our system of Boolean combinations of linear constraints does not have a solution with putative nutrient set *N*, such set of fluxes does not exist.

Notice that like the steady-state model, under the perfect data assumption, the machinery-duplicating model can produce only false positives; if it predicts growth on a putative nutrient set *N* then growth is arithmetically possible but, due to the considerations previously mentioned, may not occur in the laboratory. As with the steady-state model, under the perfect data assumption, false negatives are impossible. If the model predicts failure to grow on a putative nutrient set *N*, then it is arithmetically impossible for the organism to grow; if growth is indeed observed in the laboratory, then we must look for errors in our choice of R and B.

Also notice that the machinery-duplicating model is more heavily constrained than the steady-state model: while both models may predict false positives, and neither can predict false negatives, any nutrient set predicted by the machinery-duplicating model must necessarily be predicted by the steady-state model while in general, the converse will not be true. We formalize this idea in the following lemmas:

##### 

**Lemma 1. ***For all reaction sets*R*, biomass compound sets*B*, and subsets*N⊂T*of transportables, ifNis a machinery-duplicating nutrient set with respect to*〈R,B〉*thenNis a steady-state nutrient set with respect to*〈R,B〉.

##### 

**Proof.** If *N* is a machinery-duplicating nutrient set with respect to 〈R,B〉 then there must exist a flux *r* that satisfies the constraints generated by the machinery-duplicating model. Because the constraints generated by the steady-state model are a subset of those generated by a machinery-duplicating model, they must also be satisfied by *r*. □

##### 

**Lemma 2. ***There exists a reaction set*R*, a biomass compound set*B*, and a*N⊂T*such thatNis a steady-state nutrient set with respect to*〈R,B〉*butNis not a machinery-duplicating nutrient set with respect to*〈R,B〉.

##### 

**Proof.** Let 〈R,B〉 be defined by the hypothetical metabolic network in Example 1. We have already established that *N*={*A*,*F*} is a steady-state nutrient set with respect to this 〈R,B〉. We now show that {*A*,*F*} cannot be a machinery-duplicating nutrient set with respect to this 〈R,B〉. For a contradiction, suppose we could satisfy the constraints of the machinery-duplicating model with the flux *r*=[*r*_1_,*r*_2_]^*T*^. First, because *C*∉*N*, the net production of *C* is constrained to be non-negative, and thus *r*_1_−*r*_2_≥0. Likewise, because *B*∉*N*, the net production of *B* is constrained to be non-negative, and thus *r*_2_−*r*_1_≥0. Combining these two inequalities we can deduce that *r*_1_=*r*_2_. Furthermore, because we must make biomass compound *E* at a strictly positive rate, we have *r*_2_>0. Now the “make it if you use it” constraints come into play. Because *r*_2_>0 we are required to make *C* at a strictly positive rate and thus *r*_1_−*r*_2_>0. But this contradicts our previous deduction that *r*_1_=*r*_2_. □

Under the perfect data assumption we claim that the machinery-duplicating model is *strictly more accurate* than the steady-state model, in the following sense: Neither model can predict a false negative (Claims 1 and 2). However, the following relation exists between false positives predicted by the two models:

##### 

**Claim 3. ***Under the perfect data assumption: *

1. There exists a dataset 〈R,B〉 and N∈T where the steady-state model predicts a false positive and the machinery-duplicating model predicts a true negative; and

2. There does not exist a dataset 〈R,B〉 and N∈T where the machinery-duplicating model predicts a false positive and the steady-state model predicts a true negative.

##### 

**Justification 3.** Part (1) follows from Lemma 2 because any negative prediction by the machinery-duplicating model must be a true negative by Claim 2 and thus the positive prediction by the steady-state model must be a false positive. Part (2) follows directly from Lemma 1 as the machinery-duplicating model can never predict a positive when the steady-state model predicts a negative.

Because the machinery-duplicating model is a theoretically more accurate model than the steady-state model, we consider only the machinery-duplicating model for the rest of this paper, except briefly in Discussion, where we compare our constraint-based modeling techniques to related work.

#### *Bidirectional reactions*

The constraint systems described above handle all reactions as unidirectional. In practice, some metabolic reactions are reversible and will flow in either direction depending on the needs of the metabolic network as a whole. One way to model this situation is to replace a bidirectional reaction with a pair of complementary unidirectional reactions. This approach has the advantage of conceptual simplicity, but having a pair of reactions requires two variables rather than one. Although replacing a full range variable with a pair of non-negative variables is a common approach in naïve expositions of linear programming, SMT solvers such as Yices [10] and, indeed, modern LP solvers, handle a single full-range variable much more efficiently than two non-negative variables.

Suppose, instead that a bidirectional reaction is handled by removing the non-negativity constraint on its variable. This requires revising the growth constraint (3) to account for reactions running backward.

For some compound $C_{j}\notin N\cup B$, let *U*_*j*_ be the set of indices of reactions that have *C*_*j*_ as a reactant or a product. We now enforce a “make it if you mention it” constraint:

(4) $$\left(p_{j}>0\right)\vee \underset{i\in U_{j}}{\wedge }\left(r_{i}=0\right)$$

This expression yields a constraint that is symmetric with respect to reactants and products.

##### 

**Theorem 1. ***For bidirectional reactions, the “make it if you mention it” constraint (*4*) is equivalent to the “make it if you use it” constraint (*3*).*

##### 

*Proof.* Clearly constraint (4) is at least as strict as constraint (3). To see that it is not more strict, consider a feasible solution to a system where bidirectional reactions are represented by pairs of unidirectional reactions and the original constraint (3) is enforced on reactants. For some reaction *R* with a positive reaction rate in the original system, the new constraint (4) enforces the additional requirement that *p*_*k*_>0 for each product compound *C*_*k*_. If there is some reaction *R*^′^ with positive reaction rate that uses *C*_*k*_, then the original constraint (3) for *R*^′^ subsumes this new requirement. Otherwise, because *R* produces *C*_*k*_ with a positive rate and it is not used by another reaction, the system as a whole must produce *C*_*k*_ with a positive rate satisfying the new requirement. □

#### Simplifying the constraint-based models

The inputs for a constraint-based model can be simplified to remove compounds that will not be involved in any potential solutions. The two major classes of compounds that can be removed in this way are *Impossible* compounds and *Useless* compounds.

For clarity we define the simplification rules on a unidirectional system where any bidirectional reaction can be split into a pair of unidirectional reactions. Complementary reactions in the simplified system can then be turned back into bidirectional reactions before constructing the system of linear constraints.

A compound *C* is *Impossible* if it is not a potential nutrient and there is no reaction to make it. Such compounds can be deleted, together with any reactions that mention them. Because no reaction has *C* as a product, any reaction that mentions it must use it as a reactant. Any reaction that uses *C* as a reactant could not have a positive rate without violating the non-negativity condition for non-nutrient compounds. The deletion of reactions may enable more compounds to be recognized as impossible, so this search for impossible compounds must be iterated to fixed-point (that is, until there is no change in the set of remaining compounds).

A compound *C* is *Useless* if it has no downstream biomass compounds. The search for useless compounds proceeds by finding the complement set of *Useful* compounds. The biomass compounds are considered useful by definition. A non-biomass compound is considered useful if it is a reactant for a reaction that produces a useful compound. This test is iterated on the compounds not currently classified as useful until a fixed point is reached, such that all compounds not currently classified as useful have been checked without adding one of them to the useful set. At this point, all compounds not in the useful set are considered useless. Such compounds are eliminated from the reactions containing them, as are any reactions whose products are all useless. This leaves the possibility that a reaction will become unbalanced by losing one or more products without being deleted. However, from a constraint-solving viewpoint, this is simply the removal of a redundant non-negativity constraint.

### Computing minimal nutrient sets

We have presented a scheme for inferring whether a given set *N* of transportables is a nutrient set by checking the satisfiability of a Boolean combination of linear inequalities. For a transportable c∈T its existence in *N* can be represented by a Boolean value, *true* or *false*; likewise, the prediction of whether *N* is a nutrient set, determined by checking the satisfiability of the linear system constructed for *N*, can also be represented by a Boolean value, *true* or *false*. We are now interested in systematically generating all minimal nutrient sets with respect to our scheme and it is perhaps not altogether surprising that we can exploit well-understood technology for computing with Boolean functions to assist us.

However, the technique that we present is quite general and, mathematically, depends on just one property of nutrient sets: adding a transportable to a nutrient set produces another nutrient set. This property is called *monotonicity* and arises in our scheme because adding a transportable to a nutrient set *removes* one or more constraints from the linear system. More generally, monotonicity will arise whenever *all* negative effects (e.g., toxicity, regulation) are ignored. Note that without monotonicity, the notion of a minimal nutrient set is much more subtle.

Our technique is based on translating the problem of computing minimal nutrient sets into the language of Boolean algebra. We then use a novel algorithm for computing a representation of all minimal nutrient sets. This new algorithm is built on top of standard tools for computing with Boolean functions.

In the following subsections, we introduce all of the concepts needed and sketch the basic technique. But, the reader should be aware that obtaining reasonable runtime performance on realistic data sets, such as EcoCyc, requires a number of algorithmic refinements and implementation details (including parallelization) that are beyond the scope of this paper.

#### *Boolean functions*

In the following discussion, we denote the set {*true*, *false*} of Boolean values by **B** and the set of vectors of Boolean values of length *n* by **B**^*n*^. We now give some elementary definitions concerning functions on Booleans.

##### 

**Definition 1. ***Given a Boolean functionf:***B**^*n*^→**B***, a vectorv*∈**B**^*n*^*is animplicantoffif and only iff*(*v*)=*true*.

##### 

**Definition 2. ***A Boolean function ofnvariables,f:***B**^*n*^ → **B***, ismonotoneif and only if for anyv*∈**B**^*n*^*such thatf(v)* = *true, making a new vectorv*^′^*fromvby converting afalsecomponent totrueguarantees thatf(v*^′^*)*=*true*.

##### 

**Definition 3. ***Given a monotone Boolean functionf:***B**^*n*^→**B***, a vectorv*∈**B**^*n*^*is aprime implicantoffif and only if*

1. *vis an implicant off; and*

2. *for every vectorv*^″^*constructed from v by converting a true component to false, we have f(v*^″^*) = false.*

The method of constructing Boolean combinations of linear constraints proposed above defines a function nutset:P(T)→B that maps each subset *N* of transportables T into a *true* or *false* result, depending on whether or not the system of constraints constructed for *N* is satisfiable.

Suppose |T|=n. We represent the subsets of T by the Boolean vectors **B**^*n*^ in the following way. We pick some linear ordering on T and represent a subset N⊂T by a vector *v*_*N*_∈**B**^*n*^ where the *i*th component of *v*_*N*_ is *true* if *N* contains the *i*th member of T (under our linear ordering) and *false* otherwise.

Under this change of representation, *nutset* becomes a monotone Boolean function *nutset*:**B**^*n*^→**B**, and the minimal nutrient sets that we seek *correspond exactly* to the *prime implicants* of *nutset*.

#### Computing prime implicants

We now consider the problem of computing all the prime implicants of an arbitrary monotone Boolean function *f*:**B**^*n*^→**B**, solely by evaluating *f* on chosen inputs without making assumptions about how *f* is defined.

Suppose we have some vector *v* such that *f*(*v*)=*true*. The obvious approach to finding a single prime implicant is to systematically go through the components of *v*, setting *true* components to *false* whenever this can be done without *f* becoming *false*. This procedure turns out to be a key step in our algorithm which we call *minimization* of *v* with respect to *f*.

##### 

**Definition 4. ***Letf:***B**^*n*^→**B***be a monotone function andv*∈**B**^*n*^*be a vector such thatf(v)*=*true*. *We define the following procedure for minimizingvwith respect tof:*

•*We keep a vector variableu*=(*u*_1_,…,*u*_*n*_) *that we initialize tov*.

•*Forifrom1up tonifu*_*i*_=*true*, *then*

*Iff*(*u*_1_,…,*u*_*i*−1_,*false*,*u*_*i*+1_,…,*u*_*n*_)=*truethen setu*_*i*_:=*false, and continue with the updated vector.*

*Otherwise we continue with the value ofuunchanged.*

*Returnuas a minimization ofvwith respect tof*.

##### 

**Theorem 2. ***The result of the minimization procedure onvreturns a prime implicant off*.

##### 

*Proof.* Note that *u*=*v* initially and *v* is an implicant of *f* by definition. At each step we update the value of *u* only if the new value is also an implicant of *f* so the result of the minimization procedure must be an implicant of *f*. To see that it must be a prime implicant, suppose it were not. Thus, some component *u*_*i*_ currently set to *true* could be changed to false and the new vector would also be an implicant. But in this case we would have set *u*_*i*_ to false when it was its turn to be considered in the loop because *f* is monotone. □

Assuming that *f* is not *false* everywhere, we can find a first prime implicant by starting with the all- *true* vector and applying the minimization procedure. The tricky part is finding subsequent prime implicants.

Given a set of prime implicants of a monotone Boolean function *f*, the problem of deciding if the set is complete is known to be coNP-complete even when *f* is explicitly given in some quite natural representations [11], so an efficient algorithm for finding a next prime implicant in the general case, where *f* is only accessed by evaluation, is unlikely.

Although the problem of finding the complete set of prime implicants appears theoretically intractable, we can solve it on the instance we care about, namely, where *f* is the function *nutset*, defined by our linear constraint system instantiated from the EcoCyc dataset.

Our method is to compute successive prime implicants using minimization, where at each step we look for a new starting point based on previously found prime implicants that guarantees minimization will find a prime implicant not previously seen.

#### *Choice vectors*

Given that we have found one or more prime implicants of some monotone Boolean function *f*:**B**^*n*^→**B** we want to test if further prime implicants exist.

##### 

**Definition 5. ***Given a collection of vectorsv*_1_,…,*v*_*k*_∈**B**^*n*^, *a vectoru*∈**B**^*n*^*is achoice vectorforv*_1_,…,*v*_*k*_*iff for alli*∈{1,…,*k*}, *u*∧*v*_*i*_≠(*false*,*false*,…,*false*), *where ∧ denotes point-wise conjunction.*

Informally a choice vector is a vector that shares one or more *true* components with each of the original vectors. Let ⊏ be the partial order on **B**^*n*^ that corresponds to the subset relation and let ⊑ be its reflexive closure. We denote the vector obtained by the point-wise negation of the components of *u* by ¬*u*.

##### 

**Theorem 3. ***Given a monotone Boolean functionf*:**B**^*n*^→**B***with prime implicantsv*_1_,…,*v*_*k*_∈**B**^*n*^, *there exists another prime implicant iff there exists a choice vectoru*∈**B**^*n*^*forv*_1_,…,*v*_*k*_*such thatf*(¬*u*)=*true*.

##### 

*Proof.* Suppose there exists a choice vector *u*∈**B**^*n*^ for *v*_1_,…,*v*_*k*_ such that *f*(¬*u*)=*true*. We can get a prime implicant *v*^′^ by minimizing ¬*u*. Furthermore, *v*^′^ cannot coincide with a known prime implicant *v*_*i*_ because *u* shares a *true* component with *v*_*i*_ and thus ¬*u* has *false* in some component where *v*_*i*_ has *true* and thus *v*^′^ will have *false* because minimization never converts *false* to *true*.

In the other direction, suppose *f* has a prime implicant *v*^′^ that does not coincide with one of *v*_1_,…,*v*_*k*_∈**B**^*n*^. Now for each *v*_*i*_ there must exist some component where *v*_*i*_ has *true* and *v*^′^ has *false*, because if *v*^′^ has *true* in each component that *v*_*i*_ has *true*, either *v*^′^=*v*_*i*_ (a contradiction) or $v^{\prime }⊐v_{i}$ and thus *v*^′^ cannot be a prime implicant (another contradiction). Thus, ¬*v*^′^ is a choice vector for *v*_1_,…,*v*_*k*_. □

The problem is that for a given collection of vectors *v*_1_,…,*v*_*k*_∈**B**^*n*^ there are many choice vectors and searching among them for a choice vector *u* such that *f*(¬*u*)=*true* is prohibitively expensive.

##### 

**Definition 6. ***Given a collection of vectorsv*_1_,…,*v*_*k*_∈**B**^*n*^, *a vectoru*∈**B**^*n*^*is aminimal choice vectorforv*_1_,…,*v*_*k*_*iff*

1. *uis a choice vector forv*_1_,…,*v*_*k*_*; and*

2. *there is no*$u^{\prime }\mathrm{⊏u}$*such thatu*^′^*is a choice vector forv*_1_,…,*v*_*k*_.

##### 

**Theorem 4. ***Given a monotone Boolean functionf*:**B**^*n*^→**B***with prime implicantsv*_1_,…,*v*_*k*_∈**B**^*n*^, *if there exists a choice vectoruforv*_1_,…,*v*_*k*_∈**B**^*n*^*such thatf*(¬*u*)=*truethen there exists a minimal choice vector*$u^{\prime }⊑u$*such thatf*(¬*u*^′^)=*true*.

##### 

*Proof.* If *u* is not already a minimal choice vector then there must exist a minimal choice vector $u^{\prime }⊑u$. Every component that is *false* in *u* must also be *false* in *u*^′^. Thus evey component that is *true* in ¬*u* must also be *true* in in ¬*u*^′^. Since *f*(¬*u*)=*true* and *f* monotone it follows that *f*(¬*u*^′^)=*true*. □

Thus we can limit our search to minimal choice vectors.

Recall that a choice vector for *v*_1_,…,*v*_*k*_ has at least one *true* component in common with each of *v*_1_,…,*v*_*k*_. Let *T*_*i*_ be the set of indices of the *true* components of *v*_*i*_.

Given a vector *x*=(*x*_1_,…,*x*_*n*_)∈**B**^*n*^ we can determine if it has at least one *true* component in common with *v*_*i*_ by forming the disjunction:

$$\underset{j\in T_{i}}{\vee }x_{j}$$

and we can determine if it has at least one *true* component in common with each of *v*_1_,…,*v*_*k*_ by forming the conjunction of disjunctions:

$$g(x)=\underset{i=1,\ldots ,k}{\wedge }\underset{j\in T_{i}}{\vee }x_{j}$$

The function *g*:**B**^*n*^→**B** thus defined is necessarily monotone as no negations are involved. Thus the choice vectors of for *v*_1_,…,*v*_*k*_ correspond to the vectors *x* that make *g*(*x*)=*true* (i.e., to the implicants of *g* and the minimal choice vectors correspond to the prime implicants of *g*).

In order to compute a new prime implicant of a monotone function *f* we still need to examine the prime implicants *u* of another monotone function *g* to find one on which *f*(¬*u*)=*true*. At first sight it might appear that we have come full circle and are back where we started, trying the find the prime implicants of a monotone Boolean function. However, recall that *f* is considered to be a black box and can be accessed only by evaluating it on each input vector whereas *g* is defined as a conjunction of disjunctions formed from previously computed prime implicants of *f*. As we will see, this symbolic representation is much more amenable to prime implicant extraction.

#### Binary decision diagrams

The *Binary Decision Diagram* (BDD) is a popular data structure for representing and manipulating Boolean functions [12,13]. Although any such scheme necessarily requires exponential space on average, BDDs exploit the regularity often present in Boolean functions of interest to yield compact representations. Moreover, algorithms exist for performing many common operations on functions represented as BDDs whose running time is polynomial in the size of the input BDDs. Free BDD libraries are readily available [14,15]. The technical details of BDDs are beyond the scope of this paper; however, one important feature of a BDD is that the complete set of implicants can be recovered by tracing the paths from its root node to its *true* terminal.

Recall that the search space has been restricted to minimal choice vectors or, equivalently, the prime implicants of *g*. We can construct the BDD for *g* by incremental updates each time that we find a prime implicant of *f*. However, to find the prime implicants of *g* at any given point, we construct a new BDD for the function *pi*_*g*_:**B**^*n*^→**B** defined by

$$\begin{array}{lll} pi_{g} & \left(x_{1},\ldots ,x_{n}\right)= & \\ & g\left(x_{1},\ldots ,x_{n}\right)\wedge \underset{i\in 1,\ldots ,n}{\wedge } & \left(\neg ,x_{i},\vee ,\neg ,g\right)\left(x_{1},\ldots ,x_{i-1},\right)\\ & & \left(\left(\text{false},x_{i+1},\ldots ,x_{n}\right)\right) \end{array}$$

Intuitively, *pi*_*g*_(*x*)=*true* if *g*(*x*)=*true* and for all *x*^′^ formed by changing a *true* component in *x* to *false*, *g*(*x*^′^)=*false*. The new BDD is constructed by applying standard BDD operations to the BDD for *g*.

We can systematically enumerate the prime implicants *u* of *g* by enumerating the implicants of *pi*_*g*_ which is done by tracing the paths in the BDD for *pi*_*g*_ from the root node to the *true* terminal^d^. As soon as we find *u* such that *f*(¬*u*)=*true*, we can stop, find a prime implicant of *f* by minimizing ¬*u*, update *g* with the new prime implicant, and start over. If we cannot find such a *u* in the implicants of *pi*_*g*_ we are done.

### Nutrient equivalence classes

How can we help a biologist user interpret a collection of hundreds or thousands of computed minimal nutrient sets? At least in the case of EcoCyc, we observe that the complete collection of predicted minimal nutrient sets has a very regular structure, and that elucidating this structure yields both a compact representation of the large collection of predicted minimal nutrient sets and, in many cases in *E. coli*, a classification of nutrient compounds into equivalence classes that correspond to biological intuitions. Specifically, computed nutrient equivalence classes often contain all compounds that supply one element (e.g., sulfur sources).

#### 

**Definition 7. ***Given a collection*N*of nutrient sets, we want to capture the notion of two transportables*$c_{1},c_{2}\in T$*being equivalent ifc*_1_*can always substitute forc*_2_*in any nutrient set wherec*_2_*occurs and vice versa.*

*Formally, we say*$c_{1},c_{2}\in T$*are equivalent with respect to*N*if and only if*

1. *For all*N∈N*such thatc*_1_∈*N:*$\left(\left(N\setminus \{c_{1}\}\right)\cup \{c_{2}\}\right)\in N$*; and*

2. *For all*N∈N*such thatc*_2_∈*N:*$\left(\left(N\setminus \{c_{2}\}\right)\cup \{c_{1}\}\right)\in N$.

This relation is trivially reflexive and symmetric, and can easily be shown to be transitive. It is therefore an *equivalence relation* on the compounds occurring in members of N and can be used to factor this subset of transportables into equivalence classes where each such compound ends up in exactly one equivalence class.

For each equivalence class of compounds we can choose a *representative compound*. Given some N∈N we can form *N*^′^ by replacing each compound *c*∈*N* by the representative compound of the equivalence class of *c*. Because of the mutual substitutability of compounds within an equivalence class, *N*^′^ must necessarily be a member of N. We call *N*^′^ the *canonical form* of *N* (given our choice of representative compounds).

If we convert each N∈N to its canonical form, we will end up with many duplicates. After removing duplicates we are left with a reduced collection $\hat{N}$ of minimal nutrient sets that will likely be much smaller and more comprehensible to the biologist — especially if the representative compound for each equivalence class was chosen to be one of the more familiar compounds from those available in the class.

Of course, the question naturally arises: What is the connection between our original collection of minimal nutrient sets N and this new reduced collection $\hat{N}$ of minimal nutrient sets?

The answer is that $\hat{N}$ along with the equivalence classes we used to compute it *exactly* encode N in the following sense: If N∈N, then there must exist some $N^{\prime }\in \hat{N}$ such that *N* can be obtained from *N*^′^ by substituting for each *c*∈*N*^′^ some compound from the equivalence class of *c*. Conversely if $N^{\prime }\in \hat{N}$ and we form a set *N* by substituting for each *c*∈*N* some arbitrary compound from the equivalence class of *c* then *N* must be a member of N.

Thus, we have a very elegant compression scheme that reduces the size of our collection of predicted minimal nutrient sets and at the same time increases the comprehensibility of our results with *zero loss of information*.

### Instantiation of generic reactions

The metabolic reaction sets found in Pathway/Genome Databases such as EcoCyc include many *generic reactions* whose substrates include metabolite classes to capture the broad substrate specificity of their catalyzing enzymes. For example, EcoCyc contains four enzymes that are described as “sugar phosphatase” (E.C. 3.1.3.23), for which the official substrate is “sugar phosphate” and the product is “sugar”.

For each generic reaction, our software generates the set of corresponding *instantiated reactions* containing solely metabolite instances. For each compound class in the left and right sides of generic reactions, the software generates new potential reactions by substituting for the compound classes all combinations of instances of those classes. Relationships between a compound class and its instances are stored explicitly in the EcoCyc compound ontology. New reaction equations are added to R only when a given substitution resulted in a mass balanced equation. New reactions are not added in ambiguous cases where more than one instance has the same chemical formula.

## Results

### The *E. coli* constraint-based model

This section describes the inputs we provided to the minimal nutrient prediction algorithm to compute the minimal nutrients of *E. coli* under anaerobic conditions. The *E. coli* constraint-based model used for this work was obtained from the manually curated EcoCyc database [8]. The set of *E. coli* biochemical reactions R was taken from an EcoCyc development version, slightly beyond the 16.1 release from June 2012. We extracted all reactions whose metabolites were all small molecules, plus all reactions within metabolic pathways (a small number of which contain macromolecule metabolites such as acyl carrier protein).

Four hundred and forty one EcoCyc generic reactions with classes yielded at least one instantiated reaction. Furthermore, unbalanced reactions were removed programmatically from R. The final R used in this work consisted of 2314 (unidirectional) reactions, of which 388 were transport reactions.

To refine and correct the reactions in the model, over the course of this work, numerous changes were made to EcoCyc as a result of our analysis of executions of the minimal nutrient algorithm. They included fixing erroneous compound structures and reaction equations, adjusting the protonation state of the compounds and reactions to pH 7.3, adding missing reactions, reversing reaction directions, changing reactions from unidirectional to reversible or from reversible to unidirectional, and adjusting cell compartment assignments of reactions. In addition, we added compound instances or reclassified existing instances under appropriate classes in our compound ontology, to allow more instantiations of generic reactions to be inferred.

One interesting example was the reaction PYRUVFORMLY-RXN, which was labelled as reversible in EcoCyc, due to a literature reference describing the *in vitro* characterization of an enzyme catalyzing the reaction. We found that some false positive predictions were apparently utilizing this reaction in the physiologically implausible reverse direction. Changing PYRUVFORMLY-RXN to unidirectional, in accordance to usage of the reaction in two *in vivo* pathways, suppressed several false positive predictions and increased the overall accuracy from 67.0% to 72.5%.

A set of 111 transportable metabolites T were supplied to the algorithm. T consisted of all carbon sources from the carbon-source Biolog Phenotype Microarray plate, plus the other element sources provided on this plate [16]. T also included 16 additional metabolites: instances of those carbon sources that were classes, plus some metabolites resulting from conversions by reactions in the periplasm of supplied metabolites into metabolites that can be transported. Oxygen (*O*_2_) was not supplied as a nutrient.

We have tried running the algorithm with all metabolites transportable by known *E. coli* transporters. Some such executions have terminated, predicting approximately 8,500 minimal nutrient sets. An execution based on the current metabolic network in EcoCyc has not terminated after two months of run time. Runs larger than the 111 transportable metabolites cannot be validated because of a lack of experimental data.

The set of biomass metabolites used in our model was similar to that used in [6]. It contains 36 compounds, including the amino acids and nucleotides, and several cell-wall building blocks that lead to lipid A disaccharide. However, the lipids leading to cardiolipin have been omitted, because at this time, the generic reactions involved in those pathways could not be instantiated properly, due to a lack of appropriate compound instances.

R and T are available in Additional files 1 and 2, respectively. Additional file 3 contains the set B of biomass metabolites. Additional file 4 contains “auxiliary compounds” that must be present for the model to run, but that are not synthesized by reactions in the model, either because the reactions are unknown, or because the reactions that synthesize these compounds are beyond the scope of the model (e.g., acyl-carrier protein).

### Predicting *E. coli* minimal nutrient sets

We ran the BDD-based minimal-nutrient-set-generation algorithm using the machinery-duplication constraint model on EcoCyc data to predict at each evaluation of a *nutset* whether or not *E. coli* would grow on a given N⊂T. A total of 787 minimal nutrient sets were found (see Additional file 5) after three days of execution on a 24-core (with Hyper threading) 2.67 GHz Intel X5650 Xeon CPU-model processor.

Given the combinatorial process by which nutrient sets are constructed from individual nutrients, this abundance of minimal nutrient sets was not surprising. However, this large solution set does not lend itself to evaluation and validation of the results, especially via laboratory experiment. To facilitate human comprehension and testing of our predictions, we used the notion of equivalence between compounds with respect to a collection of nutrient sets (Definition 7, Methods) to factor the set of compounds occurring in predicted minimal nutrient sets into equivalence classes. By picking a representative compound within each such equivalence class and discarding minimal nutrient sets that contain equivalence-class members other than those chosen representatives, we obtained a reduced group of representative minimal nutrient sets from which each original minimal nutrient set could be generated by the appropriate substitution of equivalent compounds (Table 2). The reduced set of solutions is much smaller and easier to inspect than the full solution set.

**Table 2:** Grouping compounds into equivalence classes clarifies their nutrient roles

**Class**	**Element(s)**	**Compounds**
1	C	alpha-D-glucose, glycerol, D-mannose, D-glucarate, and 27 others
2	C, P	beta-D-glucose-6-phosphate,alpha-D-glucose-1-phosphate, 2 others
3	C, N	N-acetyl-beta-D-glucosamine, L-serine, adenosine, and 7 others
4	C, N	L-alanine, D-alanine, and 2 others
5	C, N	glycylproline
6	C	(R)-malate
7	C	acetoacetate
8	C	fumarate
9	C	2-oxoglutarate
10	C	acetate
11	C	formate
12	C	(S)-lactate
13	C	succinate
14	C, N	ethanolamine
15	C, N	L-proline
16	C, N	L-glutamine
17	C, N	L-glutamate
18	N	ammonium
19	C, P	sn-glycerol-3-phosphate
20	P	phosphate
21	S	sulfate

The equivalence classes of compounds generated from our original minimal nutrient sets are shown here. All the compounds in an equivalence class are interchangeable in their roles in predicted minimal nutrient sets. For example, alpha-D-glucose (class 1) can substitute for glycerol, D-mannose, and so forth. Column one shows the class’ number, column two shows the elements that we believe it provides as part of predicted minimal nutrient sets, and column three lists all or a representative part of the compounds contained within the class.

We determined 21 nutrient equivalence classes (a full listing is provided in Additional file 6), which are used in 85 reduced minimal nutrient sets (see Additional file 7). This reduction provided an approximately 9-fold decrease in the number of solution nutrient sets to facilitate review by the user.

### Evaluating predicted nutrients

We compared our predictions against published data on anaerobic *E. coli* metabolism, which were generated using Biolog Phenotype Microarray (PM) technology [16].

PMs evaluate the metabolic activity of an organism on multiple distinct sets of nutrients in parallel, allowing high-throughput analysis. Although PM technology measures respiration rather than growth, it usually represents a reasonable proxy for growth.

One important limitation of PM data is that it typically tests a single element axis at a time. For example, one PM 96-well plate tests a wide array of carbon sources while providing fixed sources of all other elements. In contrast, some of our computed minimal nutrient sets include single metabolites that are predicted to source multiple elements.

The anaerobic PM data we had access to [16] tested solely for carbon sources. We compared our computational results to these PM results as follows. Each PM well is considered to be a nutrient set *N*^*PM*^ consisting of four metabolites, each of which sources one of the elements C, N, P, or S. If an exact match of *N*^*PM*^ can be found with one of the predicted minimal nutrient sets, *N*^*pred*^, then we count this predicted nutrient as a correct prediction (true positive). Because our method predicts some nutrients that provide more than one element, we also count subset matches as true positives, i.e., *N*^*pred*^⊂ *N*^*PM*^. As an example, alpha-D-glucose-1-phosphate occurs in a predicted nutrient set together with ammonium and sulfate. However, this nutrient set does not exactly match any PM nutrient set, because every PM well in the carbon-source plate also includes phosphate as a separate metabolite. But our method predicted that alpha-D-glucose-1-phosphate can also serve as a phosphorous source, and that it is thus redundant to add phosphate explicitly to the nutrient set. By allowing subset matches, we can correctly score the *N*^*pred*^ consisting of alpha-D-glucose-1-phosphate, ammonium, and sulfate as a true positive.

If an *N*^*PM*^ demonstrated growth experimentally and matches an *N*^*pred*^, we score a true positive prediction; if no matching *N*^*pred*^ was found, we score a false negative. If *N*^*PM*^ demonstrated no growth, and did not match any predicted nutrient set, a true negative is scored; if it does match an *N*^*pred*^, then a false positive is scored. A table with all results is provided in Additional file 8.

When evaluated in this way, the overall prediction accuracy of our method was 72.5% based on 91 experimental data points (Table 3). The inconclusive 5 data points showing low growth were ignored.

**Table 3:** Our method predicted nutrients with an accuracy of 72.5% comparing to 91 experimental data points

**Source**	**Input**	**Experimental**	**TP**	**TN**	**% Total accuracy**	**FP**	**FN**
	**nutrients**	**evidence available**					
Carbon	111	91	30	36	72.5%	17	8

For each element, the table lists how many compounds were predicted to provide that element (*Input nutrients*), how many of those had experimental evidence against which they could be evaluated (*Experimental evidence available*, EV), and the results of that evaluation. A compound is a True Positive (TP) if it was predicted to provide that element and did so. A compound is a True Negative (TN) if it was predicted to *not* provide that element and actually did not. False Positives (FP) and False Negatives (FN) are also reported. The accuracy is obtained by dividing TP + TN by EV and multiplying by 100.

Six of the eight false-negative predictions are due to missing knowledge regarding the fate of the nutrients in *E. coli*. For some nutrient in these nutrient sets, no known transport reactions or consuming metabolic reactions could be found in the literature. If these six false negative predictions are removed from the comparison, the prediction accuracy of our method was 77.6%.

## Discussion

The increasing ease with which complete genomes can be sequenced should be accompanied by the ability to make predictions about the growth requirements of the corresponding organisms. We have previously shown that the metabolic network and transporter suite for a given organism can be inferred from its annotated sequence. We have shown here that using such databases to predict a large number of nutrient sets that should support growth of the organism is both possible and practical. These predictions can be distilled into a testable set of compound equivalence classes.

### Strengths and limitations of our method

As explained earlier (Claim 2, Methods), given a complete set of reactions, and a set of biomass compounds without extraneous members, our model for predicting whether an organism can grow on a putative nutrient set can produce only false positives; false negatives are impossible. Thus, when used to compute minimal nutrient sets, our method can find sets of compounds that allow the possibility of growth (based on molecule counting) but offers no guarantee that such growth can actually occur in reality. This occurs because of a number of potential negative effects on growth that our model does not attempt to account for.

Our method cannot predict the relative concentrations of nutrients, because our method does not account for enzyme reaction rates or regulation at the level of enzyme abundance and activity. Thus, some of the predicted minimal nutrient sets that work on a “parts” level by providing increasing amounts of all the correct metabolites would possibly not lead to viable growth. For example, it is known that growth of *E. coli* with glucose as the carbon source suppresses expression of several nitrogen-assimilation enzymes. As a consequence, even though the metabolic network may suggest that glucose and certain nitrogen sources should be able to work together to provide the organism’s carbon and nitrogen needs, genetic regulatory interactions mean that these combinations will prove inviable in the laboratory.

In the future, extending our method to incorporate many of these factors may be possible, but we can also learn a great deal from the differences that we see between our current minimal nutrient predictions and experimental reality.

A special and fascinating case of this difference between prediction and reality is toxicity. It is possible for a nutrient to simultaneously be a correct growth solution *and* a toxin. “The dose makes the poison,” and even typical nutrients such as glucose are naturally toxic at high concentrations. Other nutrients, however, have a surprisingly narrow gap between viability and toxicity. Thus, predicting a growth solution that is both correct and potentially difficult to apply in the laboratory is possible. The upshot of this effect is that in many cases a laboratory researcher operating without the guidance of a prediction might accidentally discard interesting, experimentally useful growth conditions based on a test that was performed using nutrient concentrations outside this “viable band”.

One key caveat about predicting “growth” for an organism based on its metabolic network arises from an increasing pool of experimental evidence that many microbes will grow only in the presence of signaling molecules. In nature, many microbes can thrive only in the presence of appropriate quorum-sensing signals from their community. When these signals are absent, they will fail to grow despite the presence of all required nutrients [17]. Although our present approach does not capture this phenomenon, a failure to grow on *any* of the predicted nutrients may be a sign that a signal should be sought.

### Related work in nutrient set prediction

The main axes of differentiation between various nutrient set prediction methods are the 

1. Mathematical model used to define growth

2. Algorithmic solving technique used to find solutions that fit that definition

3. Procedure for enumerating all possible sets of minimal growth media

#### *Defining growth*

There are different notions in the literature for how a nutrient set is defined to support growth.

The simplest definition is based on reachability — a nutrient set supports growth if there is a path from available nutrients to every biomass metabolite [18]. In this definition, special care is taken sometimes to deal with “bootstrapping” or “self-regenerating” compounds [6,18,19]. This simplified definition sets aside stoichiometric information, which significantly limits the accuracy of its predictions. However, reachability is a necessary condition for model correctness. If an experimentally validated minimal nutrient set cannot generate every biomass metabolite, then there is a gap in the metabolic model that must be fixed.

The more commonly used definition of growth is based on flux-balance analysis (FBA), which is a classical approach for performing structural (topological) analysis of metabolic networks [20]. If *M* is the stoichiometric matrix and *r* is a vector of reaction fluxes, then FBA defines *r* to be a steady state of the network if *Mr*=0. The set of reactions includes uptake reactions that encode the availability of given nutrients. Furthermore, a special reaction that uses all metabolites required for biomass production is also added to the set of reactions. In FBA, the given nutrient set is said to support growth if there is a solution *r* for reaction fluxes such that *Mr*=0 and the growth reaction has nonzero flux.

In our approach, we also use a different definition where we require a net positive production (rather than zero) for every metabolite that is involved in a reaction with nonzero flux. There are two reasons for considering this alternate formulation. First, FBA is highly sensitive to missing reactions in the metabolic network. For example, if no reactions that use a metabolite, say *D*, exist, then *Mr*=0 forces the flux on all reactions that produce *D* to be zero. We now illustrate this scenario. Recall Example 1 from Methods; here we have two reactions:

A+B→C+D,C+F→B+E

and *E* is the sole biomass compound. We now add the following exchange reactions,

→A,→F,E→

that capture the information that *A*,*F* are available as nutrients and *E* is a biomass compound that we need to synthesize. Because *D* is not consumed by any reaction, it follows that the flux on the first reaction must be zero and that all steady-state fluxes must be zero. (In other words, *r*=0 is the only solution of the constraint *Mr*=0, where *M* is the 5×5 compounds). Thus, FBA will conclude that no steady-state solutions exist because the model is missing some reactions. If we add dummy reactions that consume compounds such as *D* (that are consumed by no other reactions in the model), then FBA is more likely to generate steady-state solutions. This shortcoming of standard FBA is overcome by having a manual curation step that adds (dummy) import, export, or spontaneous, reactions [21]. The generalization from *Mr*=0 to *Mr*≥0 in our approach partly solves the problem of missing reactions. Specifically, we do not need dummy export reactions (for compound *D*, for example) because *D* can have a net positive production in a solution of our constraints.

The second reason for proposing an alternate definition of growth concerns the case when the metabolic network has cycles, a common scenario. As we claimed earlier (Claim 2, Methods), a growing and dividing cell must be able to duplicate the metabolic machinery it uses to grow on a given nutrient set, and this is not accounted for in FBA. In our approach, cycles are handled by introducing disjunctive constraints. A side effect of our solution is that each individual constraint in our approach is a disjunction of linear inequalities. In contrast, in flux-balance analysis, each individual constraint is a linear equation or linear inequality. Table 4 shows the constraints arising from reactions of the running example for FBA and our approach.

**Table 4:** Comparing constraints generated by FBA and by our approach

**cpd**	**FBA constraint**	**Our constraint**
A	−*r*_1_+*r*_3_=0	
B	−*r*_1_+*r*_2_=0	−*r*_1_+*r*_2_>0 ∨ *r*_1_=*r*_2_=0
C	*r*_1_−*r*_2_=0	*r*_1_−*r*_2_>0 ∨ *r*_1_=*r*_2_=0
D	*r*_1_=0	*r*_1_>0 ∨ *r*_1_=0
E	*r*_2_−*r*_5_=0	*r*_2_>0
F	−*r*_2_+*r*_4_=0	

For a reaction network consisting of two reactions, *r*_1_:*A*+*B*→*C*+*D* and *r*_2_:*C*+*F*→*B*+*E*, nutrients {*A*,*F*} and essential compound *E*, FBA generates the constraints in the second Column (FBA) and determines growth by maximizing *r*_5_ subject to these constraints and subject to bounds on influx of nutrients, 0≤*r*_3_≤*r*_3*max*_ and 0≤*r*_4_≤*r*_4*max*_. We generate four constraints, shown in the third column, out of which three are disjunctive. Note that we do not use the dummy reactions *r*_3_:→*A*, *r*_4_:→*F* and *r*_5_:*E*→.

Although the FBA approach does not account for possible problems induced by cycles, it seems to give good results. An interesting problem for future work is to understand what features of a metabolic network suppress the effects of cycles on the space of solutions.

#### Solving technique

When plain reachability is used to define growth, a simple forward propagation procedure — based purely on qualitative reasoning — suffices for deciding if a given medium supports growth [18]. Such a procedure is efficient, but makes an unrealistic assumption that reactants of a reaction are not used up when that reaction is used.

Flux-balance analysis uses standard Linear Programming (LP) solvers for finding the maximum flux for the biomass generation reaction subject to the constraint *Mr*=0. In our approach, we generate disjunctions of linear constraints, and hence we cannot use LP solvers. We instead use modern and highly efficient solvers, called Satisfiability Modulo Theory (SMT) solvers [22-25]. Not only do SMT solvers handle more general constraints, they also support a rich interface that enables incremental addition and retraction of constraints. This feature allows the exhaustive search for minimal nutrient sets to be made more efficient, by sharing computation between the individual evaluations of *nutset*.

#### Enumerating all nutrient sets

The problem of enumerating all minimal nutrient sets has not been widely studied. Handorf et al. [18] and Cottret et al. [19] are the only works that attempt to analyze all minimal nutrient sets. Handorf et al. [18] state that enumerating all minimal sets is “impossible” and hence, a random (biased) sampling process is used to enumerate some (at most 1000) of the minimal nutrient sets. The sampled minimal nutrient sets are used to perform additional analysis, such as identifying exchangeable resource metabolites and essential clusters. The authors have to manually pick threshold values for classification and to also manually merge equivalence clusters [18].

Cottret et al. [19] perform a straightforward exhaustive enumeration of possible nutrient sets by building an (exponentially large) tree representing the backward reachable sets starting from the target biomass compounds. Stoichiometry information is not used in this process and reactants are not “used up” when they are fired; for example, given the two reactions 2*A*→*B*, *B*→*A*, they will conclude that the network can synthesize *B* starting from an empty bag of nutrients. The scalability of the approach on large reaction networks, such as from EcoCyc, is a concern: Cottret et al. [19] show that the forward reachability can be performed on large networks, but the enumeration of all nutrient sets is done on only small networks.

Feist et al. [21] and Maranas et al. [26] use FBA-based techniques to determine all carbon, nitrogen, phosphorous, and sulfur sources that could support simulated growth. But rather than considering all minimal nutrient sets, their method selects a “seed” minimal medium and then varies one of its nutrient sources (carbon/nitrogen/phosphorous/sulfur) at a time, and predicts if growth is possible. This approach, which we call single-element variation, assumes that the choice of nutrient source for a given element (C, N, P, or S) is independent of the other choices (i.e., that nutrient sources for a given element can always substitute for one another). Seeing that this assumption might be false is easy, for example, consider a trivial metabolic system involving only carbon and nitrogen. Suppose we have two carbon sources *c*_1_ and *c*_2_ and two nitrogen sources *N*_1_ and *N*_2_, with compound *M* representing biomass. Consider the reactions:

$$\begin{array}{ll} C_{1}+N_{1}\to M & \;\\ C_{2}+N_{2}\to M & \; \end{array}$$

Clearly, *c*_1_ and *c*_2_ cannot substitute for one another. The single-element variation method might choose nutrient set {*C*_1_,*N*_1_} as its seed nutrient, and vary the *N* source to produce nutrient set {*C*_1_,*N*_2_}. If this nutrient set failed to support growth, the method would erroneously conclude that *N*_2_ could never function as a nitrogen source.

Another problem with the single-element variation method is that it assumes exactly one nutrient is needed for each element, which might be false. Consider a metabolic system that is configured such that one set of nutrients can supply nitrogen to amino acids only, and an orthogonal set of nutrients can supply nitrogen to nucleotides only, with no possible flow of nitrogen between the amino acids and the nucleotides.

One might argue that such metabolic systems have never been observed in the natural world, so why should we build algorithms to detect them? We argue the converse: that if we do not build algorithms to detect them, we will never discover them from sequenced genomes, and given the incredible diversity of nature, such systems may well exist. For example, many genome sequences are in hand for parasitic microbes that have lost major components of their metabolic machinery. By using a novel algorithm built on top of a classical data representation (Binary Decision Diagrams), we can systematically search an otherwise intractably large space without making any assumptions about the independence of elemental sources. The full pool of 236 known *E. coli* transportable instance metabolites would expand to on the order of 10^7^ potential four-compound combinations (corresponding to sources of C, N, P, and S). If we consider that two distinct compounds might source C together, on the order of 10^9^ five-compound combinations would be obtained. Since in general an organism might require more than one source of a given element, we cannot decide *a priori* the upper bound on the number of nutrients to consider.

Other efforts to use genome-scale metabolic models to determine minimal nutrient sets include [27-30]. Each of these efforts uses an FBA approach to check viability of nutrient sets, only selectively varying single nutrients once a starting minimal nutrient set has been found. The range of variation is mainly constrained to compounds available in Biolog phenotype microarrays, so the predictions can be readily checked against experimental results.

In contrast to all work described above, we present a technique for computing *all* minimal nutrient sets. We demonstrate that the computation is feasible for large genome-scale models.

Moreover, our approach for enumerating all nutrient sets is generic — it is independent of the underlying definition of growth and the solver used.

Unlike FBA, our approach does not need an objective function because it is not based on solving an optimization problem.

The various methods described above, including our approach, predict growth on minimal nutrient sets based on structural analysis of the metabolic network. All these methods are limited by the accuracy of the compounds and reactions modeled in the metabolic network, by their list of transportable nutrients, and by the specification of biomass compounds.

## Conclusions

We have described a method for computing alternative minimal growth media for an organism based on its metabolic network and transporter complement. The method combines linear constraint solving with binary decision diagrams. Whereas previous approaches to this problem did not consider all possible combinations of nutrients, our method does consider all such possibilities. Previous approaches assumed that all element sources are independent from one another, and that one source of each element only is required, whereas we show that in general these assumptions are invalid. Science is unlikely to detect organisms whose metabolic networks violate those assumptions unless we have computational methods that do not depend on those assumptions. A key aspect of our approach is the machinery-duplicating constraint, namely that all metabolites used in active reactions must be produced in increasing concentrations to prevent cell divisions from diluting these metabolites to the point that they are not available to the cell’s metabolic network. We validated our method by predicting alternative minimal nutrient sets of *E. coli* K–12 MG1655 under anaerobic conditions. These minimal nutrient sets were predicted with 72.5% accuracy as evaluated by comparison with data from 91 growth experiments.

### Future goals and methods

The method that we present in this paper must next be applied beyond *E. coli* to aid researchers who are trying to study uncultivatable pathogens and environmentally sampled organisms, and to develop effective synthetic biology platforms. The ability to rapidly sequence and annotate such research targets must necessarily be complemented by the ability to quickly address potentially enormous research challenges such as “How do we grow this organism?” into tractable questions.

Clear areas for future enhancements to our method exist, beginning with developing a better understanding of the source(s) of the differences between our predictions and biological reality. The goal will be to develop tools to identify when discrepancies represent a problem with the method or a potential area of new study. These improvements will be built on the back of enhancements that make the method itself more computationally efficient, opening up the opportunity to include knowledge of regulation, metabolite concentrations, and other factors that will become more readily available as new high-throughput methods are developed.

## Endnotes

^a^Note that this cannot be formulated as a theorem since a theorem can only state properties about models of the real world rather than about the real world itself. Justification of claims in this section tacitly rely on a model of the world that is often implicitly assumed in biology and where the notion of discrete biochemical reactions makes sense. In particular organisms are assumed to be composed of molecules and a molecule is considered to be a discrete assemblage of atoms. Molecules are only transformed by biochemical reactions and those reactions must be balanced with respect to counts of each kind of atom. Atoms themselves are assumed to be indivisible, immutable, and conserved. We will maintain this fiction for the rest of this paper.^b^We make the simplifying assumption that a given cell divides into two daughter cells that are identical at the molecular level. Of course in practice this is extremely unlikely; however, we really only require that the daughter cells are *sufficiently similar* in terms of their molecular composition. This vague notion of sufficiently similar could be made precise by the development of a formal mathematical measure of fission similarity based on the exact molecular composition of the cells in question and then we could formally prove the validity of our model for organisms that have fission similarity above a certain threshold. However, since determining the exact molecular composition of a given cell is well in advance of current experimental technique, the development of such a mathematical theory at the present time seems to us to be superfluous, and is certainly beyond the scope of this paper.^c^The word *feasibility* is the standard terminology in the field of Linear Programming, while the word *satisfiability* is the standard terminology in the field of Computational Logic, where SMT solvers were developed.^d^Because *pi*_*g*_ encodes the prime implicants of a monotone function it can never happen that we have two prime implicants that differ only on the value of a single component. Consequently, every variable must occur as the label for some node on every path from the root node to the *true* terminal, which slightly simplifies the extraction of the implicants of *pi*_*g*_.

## Competing interests

The authors declare that they have no competing interests.

## Authors’ contributions

SE designed and implemented the minimal-nutrients algorithm and authored sections of the manuscript. MK contributed to the design of the algorithm; he ran the algorithm and analyzed its results; he updated the EcoCyc metabolic network to incorporate new information that was found as a result of analyzing algorithm runs; he authored sections of the manuscript. AGS contributed to the design of the algorithm and the analysis of its results, and authored sections of the manuscript. AT contributed to the design of the algorithm and authored sections of the manuscript. IMK contributed to the analysis of runs and updating of the EcoCyc metabolic network. CT contributed to the design of the algorithm. PDK contributed to the design of the algorithm and the analysis of its results, and authored sections of the manuscript. All authors read and approved the final manuscript.

## Supplementary Material

Additional file 1 **The set of EcoCyc biochemical reactions, **R**.** Click here for file

Additional file 2 **The set of transportable compounds, **T**.** Click here for file

Additional file 3 **The set of biomass metabolites, **B**.** Click here for file

Additional file 4 The set of auxilliary compounds that must be present for the model to run. Click here for file

Additional file 5 All minimal nutrient sets computed by our program. Click here for file

Additional file 6 The nutrient equivalence classes. Click here for file

Additional file 7 The reduced minimal nutrient sets. Click here for file

Additional file 8 Growth status for each compound. Click here for file
